# Expression and Purification of Chaperone-Active Recombinant Clusterin

**DOI:** 10.1371/journal.pone.0086989

**Published:** 2014-01-23

**Authors:** Rebecca A. Dabbs, Mark R. Wilson

**Affiliations:** Illawarra Health and Medical Research Institute, School of Biological Sciences, University of Wollongong, Wollongong, New South Wales, Australia; UMCG, Netherlands

## Abstract

Clusterin was the first described secreted mammalian chaperone and is implicated as being a key player in both intra- and extracellular proteostasis. Its unique combination of structural features and biological chaperone activity has, however, previously made it very challenging to express and purify the protein in a correctly processed and chaperone-active form. While there are multiple reports in the literature describing the use of recombinant clusterin, all of these reports suffer from one or more of the following shortcomings: details of the methods used to produce the protein are poorly described, the product is incompletely (if at all) characterised, and purity (if shown) is in many cases inadequate. The current report provides the first well validated method to economically produce pure chaperone-active recombinant clusterin. The method was developed after trialling expression in cultured bacterial, yeast, insect and mammalian cells, and involves the expression of recombinant clusterin from stably transfected HEK293 cells in protein-free medium. The product is expressed at between 7.5 and 10 µg/ml of culture, and is readily purified by a combination of immunoaffinity, cation exchange and size exclusion chromatography. The purified product was shown to be glycosylated, correctly proteolytically cleaved into α- and β-subunits, and have chaperone activity similar to that of human plasma clusterin. This new method creates the opportunity to use mutagenesis and metabolic labelling approaches in future studies to delineate functionally important sites within clusterin, and also provides a theoretically unlimited supply of recombinant clusterin which may in the future find applications in the development of therapeutics.

## Introduction

Clusterin (CLU) was the first described extracellular mammalian chaperone [1] and can interact with a wide variety of misfolded proteins to stabilise them in a soluble form, until the bound protein can be re-folded or degraded [1], [2], [3]. CLU binds preferentially to intermediate folding states of proteins that are slowly aggregating, long-lived and precipitation bound [4], and can inhibit both amorphous and amyloid pathways of aggregation [5], [6], [7]. *In vivo*, in a rat model, complexes formed between CLU and misfolded client proteins are rapidly cleared by hepatocytes and degraded within lysosomes; receptor-mediated endocytosis by scavenger receptors is implicated in the clearance [2]. It has been proposed that CLU is one of a family of extracellular chaperones that act to clear extracellular body fluids of toxic aggregating proteins, and thus plays a pivotal role in extracellular protein homeostasis (proteostasis) [8]. As a case in point, it was recently shown that changes in the clusterin gene can pose a very significant risk factor in Alzheimer’s disease [9], [10] and that CLU reacts with a variety of Aβ oligomers, ranging from monomers to 50-mers, to inhibit their aggregation and toxicity [11]. Furthermore, under conditions of endoplasmic reticulum (ER) stress, CLU can retrotranslocate from the secretory pathway into the cytosol where it can also contribute to the maintenance of intracellular proteostasis [12], [13]. Thus CLU is currently unique in that it is the only chaperone known to participate in processes of both intra- and extra-cellular proteostasis.

Despite this unique position, and considerable interest in CLU for its roles in proteostasis, many serious protein misfolding diseases and cancer [8], very little is known about the structure-function relationships of the molecule. There have been no crystal structure determinations for the protein, and only limited analyses by mass spectrometry [14], [15] and NMR [4]. What little is known about the structure comes largely from predictions based on amino acid sequence analyses [16]. Inferences about the potential location of CLU binding sites for the low density lipoprotein receptor-related protein 2 (LRP-2; glycoprotein 330/megalin) receptor and misfolded proteins have been drawn from fortuitously truncated versions of recombinant CLU (rCLU) expressed in yeast [17] but are yet to be clearly identified. Similarly, other regions of the protein that may be critical in the chaperone action are currently unknown.

CLU is translated on cytosolic ribosomes with a 22-mer ER signal sequence which is cleaved once the protein enters the ER, to produce a 427 amino acid polypeptide with a predicted mass of 50,062 Da. The protein is subsequently internally proteolytically cleaved in the Golgi to generate the α- and β-chains which are joined by 5 disulfide bonds [18]. The protein is also heavily glycosylated at multiple sites, to give 17–27% carbohydrate by mass, which results in SDS-PAGE in the protein having an apparent mass of 75–80 kDa (the actual mass is approximately 58–63 kDa; [15]). Thus CLU undergoes extensive eukaryote-specific post-translational modification. This structural complexity, together with the propensity of CLU to form very stable complexes with other proteins that misfold during extended cell culture [14], [19], make CLU particularly challenging to generate as a recombinant product. These challenges have previously impeded conventional mutagenesis approaches and made other structural determinations of the molecule difficult.

Clearly bacteria are unable to perform the proteolytic cleavage of CLU into α- and β-chains, or to glycosylate the protein. It is known that human plasma CLU maintains chaperone action when enzymatically deglycosylated [14], however, it is unknown whether the α-β cleavage is required for chaperone activity. Therefore, bacterial expression systems (which are fast and relatively cheap) might in theory be capable of producing chaperone-active rCLU, although eukaryotic systems offer the advantage of fuller post-translational modifications.

When we previously expressed rCLU in Sf9 *Spodoptera frugiperda* (insect) cells, the product was not proteolytically cleaved into α- and β-chains, had variable levels of glycosylation and a low yield (F Dawes, unpublished data). Another group used baculovirus to express rCLU in H5 *Trichoplusia ni* (insect) cells, which resulted in an under-glycosylated, non-secreted product that had not been post-translationally cleaved into mature α- and β-chains [20], indicating that these may be common problems when expressing rCLU in insect cells. We also previously expressed a glycosylated, disulphide-bonded α-β clusterin dimer in the yeast *Pichia pastoris*, however, the product was partially proteolytically degraded [17].

Other groups have expressed rCLU in *E. coli* [21], yeast [22] and various mammalian cell lines [23], [24], [25]. In these studies, however, rCLU was not well characterised; typically a Western blot was performed to verify the identity of the product (although in some cases this was not shown), and in most cases SDS-PAGE analyses were not shown nor the purity of the sample described. Methods used to purify rCLU are also poorly described in these publications, making it hard to reproduce and/or validate the methods. Furthermore, critically, none of these previous studies attempted to verify that the product was chaperone-active.

Collectively, the available information raises questions about whether any previously published study has in fact produced ‘pure’ appropriately post-translationally processed rCLU, let alone a functionally active product. Therefore, to enable future progress in understanding the structure-function relationships of this important chaperone and its roles in proteostasis and disease, there was an urgent need to develop a successful method to produce pure, chaperone-active rCLU; development of a suitable method involving expression from transfected HEK293 cells cultured in protein-free medium is described below.

## Methods

### Ethics

Human blood was obtained as a gift from Wollongong hospital and ethics approval for this was obtained from the Human Ethics Committee at the University of Wollongong (HE02/080). All donors were being treated by routine venepuncture for polycythemia or other conditions and gave their consent in writing prior to blood collection.

### SDS-PAGE

The purity and molecular weight of protein samples were assessed using SDS-PAGE. Samples were prepared in 1X sample buffer (60 mM Tris pH 6.8, 1% (w/v) SDS, 10% (v/v) glycerol, 0.01% (w/v) bromophenol blue) and heated at 100°C for 5 min prior to loading on the gel. Samples were loaded onto a 10% (v/v) SDS-PAGE gel and electrophoresed at 120 V in a Hoefer™ SE 250/260 SDS-PAGE apparatus (GE Healthcare, Chalfont St Giles, UK) and subsequently stained in Coomassie Blue (0.2% (w/v) Coomassie Blue R250, 40% (v/v) methanol, 10% (v/v) glacial acetic acid in distilled water (dH_2_O)) and destained in 40% (v/v) methanol, 10% (v/v) glacial acetic acid and 50% (v/v) dH_2_O.

### Immunoblotting

For Western blots to detect CLU, samples were electrophoresed on a 10% SDS-PAGE gel followed by electrophoretic transfer using a Trans-blot (Bio-Rad, Hercules, CA, USA) apparatus. Following protein transfer, the nitrocellulose membrane (Pall Corporation, Pensacola, FL, USA) was blocked overnight at 4°C in HDC/PBS (1% (w/v) heat denatured casein +0.01% (w/v) thimerosal in phosphate buffered saline (PBS): 137 mM NaCl, 2.7 mM KCl, 7.9 mM Na_2_HPO_4_, 1.5 mM KH_2_PO_4_, pH 7.4). After blocking, a 1∶1 mixture of undiluted G7 and 41D hybridoma culture supernatants [26] was added to the membrane and incubated for 1 h at 37°C with shaking prior to washing. Between each incubation the membrane was washed with 0.1% (v/v) Triton X-100 (Tx-100) in PBS three times followed by a rinse with PBS. The secondary antibody used was either goat-anti-mouse-HRP or sheep-anti-mouse-HRP (both from Millipore, Billerica, MA, USA) diluted 1∶1,000 in HDC/PBS and incubated as above. An enhanced chemiluminescence (ECL) kit (Supersignal Western Pico substrate working solution; Thermo Scientific, Rockford, IL, USA) and Amersham Hyperfilm ECL (GE Healthcare) were used for development, as per the manufacturer’s instructions. For immuno dot blot analyses of HEK293 cell culture supernatants, aliquots from a binary dilution series of the sample were spotted onto nitrocellulose membrane. For comparison, known concentrations of wild type CLU purified from human plasma (wtCLU; see below) were also spotted onto the membrane. After air drying, the membrane was blocked and probed for CLU as described above.

### Expression of Recombinant CLU in Escherichia coli

*E. coli* was routinely cultured in Luria Bertani (LB) broth (10 g/l tryptone, 5 g/l yeast extract, 171 mM NaCl in dH_2_O), or on LB agar (LB broth supplemented with 15 g/l agar) plates. Electrocompetent *E. coli* were transformed with a pDEST42 expression vector (Invitrogen, now Life Technologies™; Carlsbad, CA, USA) containing human CLU cDNA (Uni-Prot P10090-1) using a Bio-Rad Gene Pulser at 2.5 KV, 25 µFD and 200 Ω, and subsequently cultured in the presence of 100 µg/ml ampicillin. Overnight starter cultures were used to inoculate 1,000 ml of pre-warmed LB broth containing 100 µg/ml ampicillin. The culture was grown at 37°C with shaking at 200 rpm until an optical density of 600 nm (OD_600_) of 0.8 was reached before inducing expression with 0.3 mM IPTG and shaking at 200 rpm overnight at 24°C. Cells were pelleted by centrifugation at 5,000×*g* for 15 min at 4°C.

### Purification of Recombinant CLU Expressed in E. coli

All bacterially expressed recombinant CLU (b-rCLU) was found in inclusion bodies; to extract b-rCLU, the bacterial pellet was resuspended in 5 ml lysis buffer (300 mM NaCl, 46.6 mM Na_2_HPO_4_, 3.4 mM NaH_2_PO_4_, 0.75 mg/ml lysozyme, 10 U/ml DNase 1, 1 mM PMSF; pH 8.0) per gram of pellet. During resuspension a Complete® Protease Inhibitor Cocktail tablet (Roche Diagnostics Australia; Castle Hill, NSW, Australia) was added. The suspension then underwent three freeze/thaw cycles using liquid nitrogen and a 37°C water bath respectively. The solution was pelleted at 10,000×*g* for 30 min and the supernatant discarded. The pellet was resuspended in 25 ml of wash buffer (300 mM NaCl, 46.6 mM Na_2_HPO_4_, 3.4 mM NaH_2_PO_4_, 0.5% (v/v) Tx-100; pH 8.0) and centrifuged at 25,000×*g* for 30 min, this step was repeated four times to wash away any proteins that may have non-specifically adsorbed to the hydrophobic inclusion bodies. The pellet was next resuspended in 5 ml of solubilisation buffer (300 mM NaCl, 46.6 mM Na_2_HPO_4_, 3.4 mM NaH_2_PO_4_, 8 M urea, 10 mM dithiothreitol (DTT), pH 8.0), gently shaken overnight at 4°C to dissolve, and then centrifuged for 30 min at 25,000×*g* to remove any remaining insoluble components. Solubilised inclusion bodies were fractionated on a Superose™ 6 10/300 column (GE Healthcare) in solubilisation buffer (containing 5 mM DTT); fractions identified by SDS-PAGE as containing b-rCLU (apparent mass of ∼50 kDa), with minimal high molecular weight (HMW) contaminants, were combined for further use.

A variety of attempts were undertaken to produce a soluble form of b-rCLU in the absence of reducing agents; in most cases the removal of urea and DTT from the solution resulted in bulk protein precipitation (Figure S1). Briefly, these methods included: a) direct dilution of b-rCLU dropwise into a refolding buffer (50 mM NaH_2_PO_4_, 300 mM NaCl, 1 mM DTT, 10% (v/v) glycerol, 0.1% (w/v) Az, pH 8), which has previously been used to refold rCLU fragments [19]; b) direct dialysis of b-rCLU in solubilisation buffer against PBS, and c) step-wise dialysis against decreasing concentrations of urea, using 1 M increments from 6 M to 1 M urea (i.e. 5 changes), followed by dialysis against 0.5 M and then 0.25 M urea in PBS before dialysis against 3 changes of PBS/Az (PBS containing 0.1% (w/v) sodium azide). Only this last method produced soluble b-rCLU, however this product rapidly and progressively formed HMW aggregates, suggesting that it was not folded correctly. Therefore reduction and alkylation was also undertaken to determine if this could prevent the progressive formation of these HMW species. Briefly, strong reducing conditions (50 mM DTT for 18 h) were found to be necessary to dissociate the HMW aggregates formed after the removal of urea and DTT (as determined by size exclusion chromatography (SEC; data not shown)) followed by cysteine capping or alkylation of free thiol groups. After reduction, the sample was passed over a PD10 column (GE Healthcare) equilibrated with PBS +3 mM EDTA to remove DTT, and fractionated into the wells of a quartz 96 well plate. The wells with the highest absorbance at 280 nm (A_280_) were pooled and a) capped with a 100X molar excess of L-cysteine, or b) alkylated with 50 mM of either iodoacetic acid (IAA), iodoacetamide (IAM), or N-ethylmaleimide (NEM). All reactions were carried out overnight at 37°C with shaking.

### Expression of Recombinant CLU Secreted by HEK293 Cells

HEK293 cells (ATCC, Manassas, VA, USA) were stably transfected with the vector pRcCMV containing human CLU cDNA (Uni-Prot 10090-1) using lipofectamine (Life Technologies™) following the manufacturer’s protocol. After transfection the cells were grown in DMEM:F-12 containing 10% (v/v) foetal calf serum (FCS) and 600 µM geneticin. To initially confirm secretion of mammalian cell-expressed recombinant CLU (m-rCLU), supernatant was collected from the HEK293 transfectants (this contained 7.5–10 µg/ml of m-rCLU, estimated by immuno dot blot; data not shown). For large-scale expression, 175 cm^2^ flasks were seeded to 50% confluency with cells suspended in 35 ml DMEM:F-12 medium supplemented with 5% (v/v) FCS and incubated for 16–24 h to allow cells to adhere (cells typically reached 60–80% confluence during this time). The medium containing FCS was subsequently removed, the cell monolayer gently rinsed twice with DMEM:F-12 to remove any remaining traces of FCS, and 35–50 ml of unsupplemented DMEM:F-12 added to the flask. Cells were cultured for a further 7–10 days without changing the culture medium, after which the supernatant was centrifuged at 300×*g* for 5 min to pellet dead cells and debris. The content of m-rCLU in the clarified cell culture supernatant was tested by both immunoblot and immuno dot blot procedures.

### Purification of m-rCLU and wtCLU

Transfected HEK293 culture supernatant was supplemented with Complete® protease inhibitor and filtered through a 0.45 µm cellulose nitrate filter (Sartorius Stedim Biotech, Goettingen, Germany). Human blood containing 10 mM sodium citrate was kindly donated by Wollongong hospital (Wollongong hospital pathology unit, NSW, Australia); plasma was prepared by centrifuging the blood at 1,300×*g* for 30 min at 4°C. Complete® protease inhibitor was added and the plasma then filtered through (i) a GFC glass fibre filter (MicroAnalytic Products Inc., Mountain Lakes, NJ, USA) to remove any large debris and clots that may have formed, and then (ii) a 0.45 µm cellulose nitrate filter. A 5 ml G7 anti-CLU monoclonal antibody column [16] was connected to an Econo pump system (Bio-Rad) and equilibrated in filtered PBS/Az. The supernatant or plasma was then pumped over the column (at 0.5 ml/min or less) to allow CLU to bind, followed by washing with (i) ∼50 ml PBS/Az, then (ii) ∼50 ml of 0.5% (v/v) Tx-100 in PBS (to remove any bound lipids), before re-equilibrating in PBS/Az. Next, 200 mM sodium acetate, 0.5 M NaCl, pH 5 was passed over the column to remove non-specifically bound material. Finally, specifically bound material was eluted using 2 M GdHCl in PBS and the column immediately re-equilibrated with PBS/Az for storage at 4°C. Fractions containing the GdHCl eluate were combined and dialysed against two changes of 20 mM MES, pH 6.0. A 1 ml HiTrap™ SP XL cation exchange column (GE Healthcare) was equilibrated in 20 mM MES, pH 6.0, the dialysed fraction loaded and the flow-through (containing CLU) collected. The bound ‘contaminating’ proteins, typically >250 kDa in size were eluted from the cation exchange column with 20 mM MES, pH 6.0, containing 1 M NaCl. An additional ‘clean up’ step of SEC on a Superose™ 6 10/300 column, as described below, was undertaken to remove remaining HMW contaminating proteins.

### Circular Dichroism Spectroscopy

A Jasco Model J-810 (Jasco, Easton, MD, USA) spectropolarimeter linked to a CDF-426S/L Peltier system (Jasco) was used to acquire circular dichroism (CD) data. Far-UV (180–250 nm) CD studies were undertaken to compare the secondary structure of proteins. Samples were analysed using a 1 mm CD cell, acquiring spectra at a sensitivity of 100 millidegrees and a bandwidth of 1 nm. The scanning mode was continuous at 50 nm/min and 6 data sets were accumulated before the average was displayed.

### SEC Analysis

Proteins were loaded onto a Superose™ 6 10/300 column equilibrated in PBS/Az using an ÄKTA FPLC system at 0.3 ml/min and the A_280_ of the eluate monitored continuously. Mass standards were from a commercial HMW calibration kit (GE Healthcare). All buffers and samples were filtered (0.45 µm) before use.

### Chaperone Function Assays

Client proteins were induced to amorphously precipitate using either heat or reductive stress; in all cases, assays were performed in flat bottom 384 well plates (Greiner Bio-One; Kremsmunster, Austria) with a final volume of 50–100 µl/well. Protein aggregation was continuously measured as turbidity (A_360_) using a FLUOstar OPTIMA plate reader (BMG Labtech; Ortenberg, Germany). The putative chaperone proteins (and control proteins) being tested were added at different concentrations to determine whether they could inhibit client protein precipitation. As the precise molecular mass of rCLU was not known (although it should be similar to wtCLU), mass ratios were used to compare the chaperone efficacy of rCLU versus wild type CLU purified from human plasma (wtCLU; see below). The chaperone activity of b-rCLU was tested against bovine serum albumin (BSA) and glutathione-S-transferase (GST), using the following conditions: BSA (1.5 mg/ml) was reduced by incubation (without shaking) for 16 h at 37°C in PBS/Az containing 20 mM DTT, and GST (500 µg/ml) was heated for 3–4 h at 60°C in PBS/Az. The chaperone activity of m-rCLU was tested using 3 client proteins: BSA (as described above), citrate synthase (CS) and creatine phosphokinase (CPK). CS and CPK were heated for 3–4 h at 43°C under the following conditions (with A_360_ readings taken every 1 min); CS (200 µg/ml) in TE buffer (50 mM Tris, 2 mM EDTA, pH 8) with 3 s double orbital shaking (1 mm shaking width, 600 rpm) after each read; CPK (1.12 mg/ml) in PBS/Az with 10 s double orbital shaking (1 mm shaking width, 600 rpm) after each read. BSA and ovalbumin (OVA) were used as non-chaperone control proteins.

## Results

### Characterisation of rCLU Expressed in E. coli

**Figure 1:**
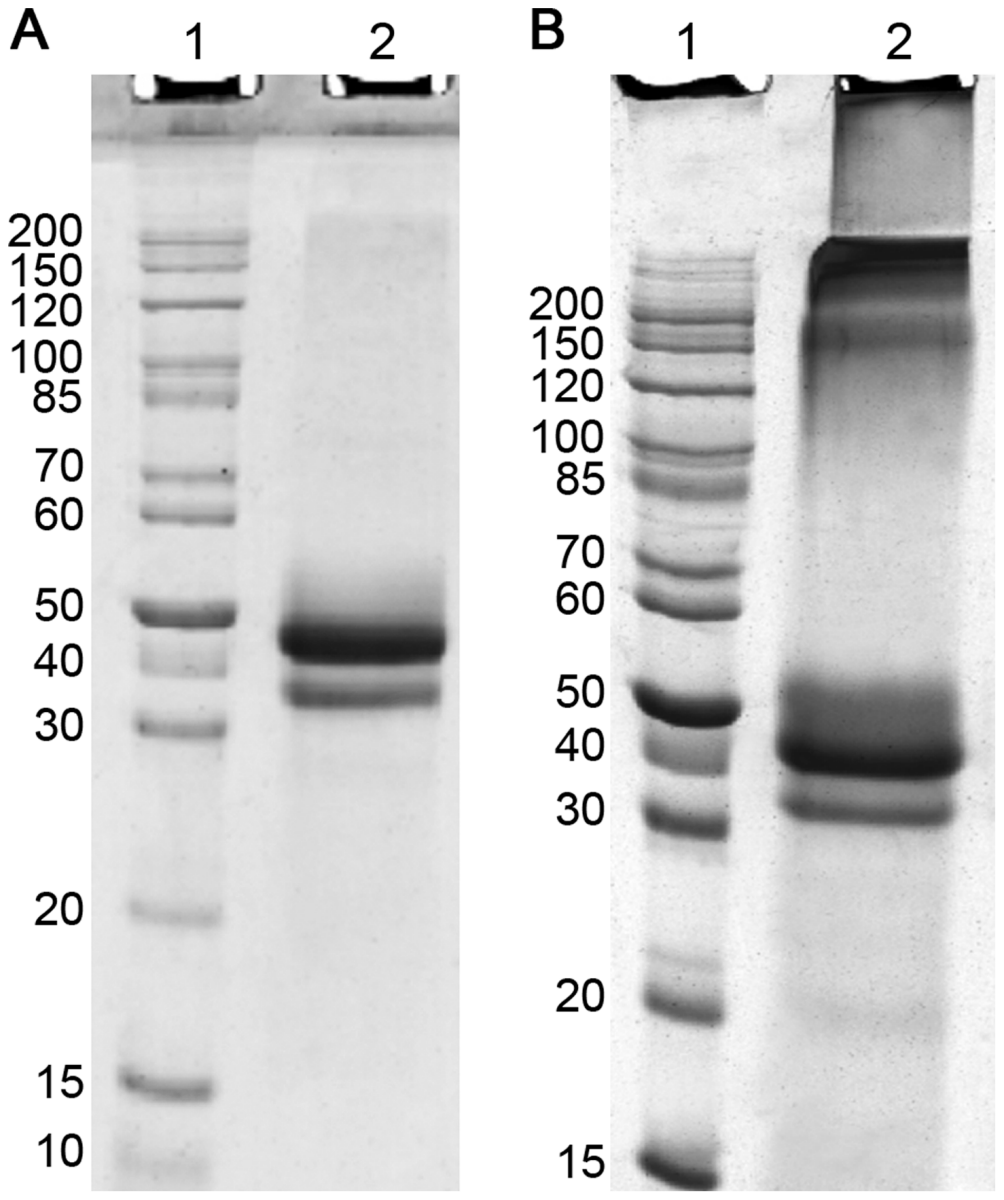
Images of Coomassie blue stained SDS-PAGE gels analysing b-rCLU. (A) Lane 1: Molecular weight markers with size indicated in kDa. Lane 2: Sample of b-rCLU after SEC purification in solubilisation buffer containing 8 M urea and 5 mM DTT. (B) Lane 1: Molecular weight markers with size indicated in kDa. Lane 2: Sample of b-rCLU after step-wise dialysis into PBS/Az.

**Figure 2:**
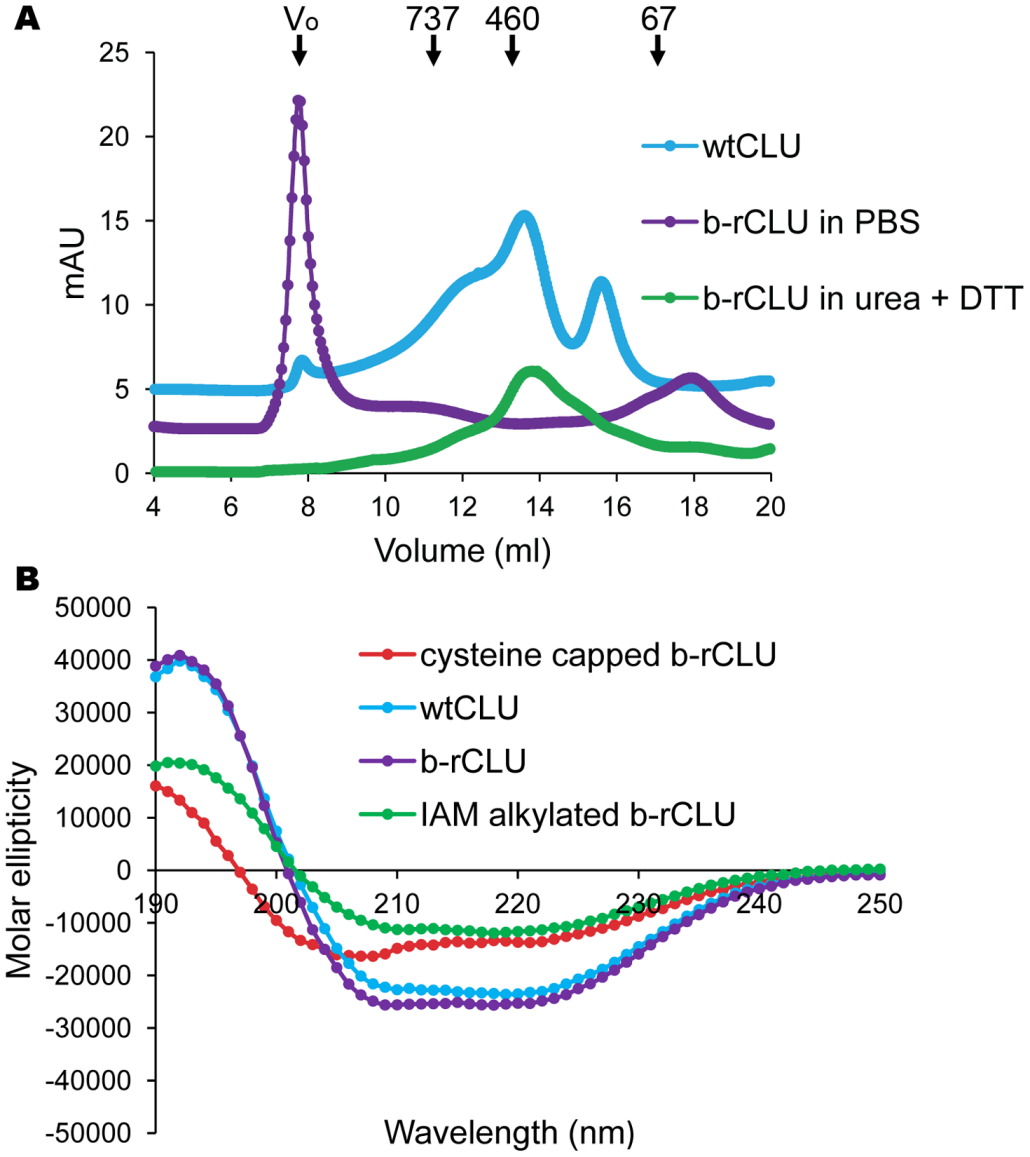
SEC and circular dichroism (CD) analyses of b-rCLU and wtCLU. (A) A_280_ traces of SEC separations performed using a Superose™6 10/300 SEC column. The positions and masses (in kDa) of molecular weight markers are indicated by labelled arrows; the exclusion limit of the column (V_o_) ≥4×10^7^ Da. For visual clarity the traces for b-rCLU (after step-wise dialysis into PBS) and wtCLU (in PBS) are deliberately vertically offset from that of b-rCLU fractionated in solubilisation buffer containing 8 M urea and 5 mM DTT. (B) CD plots represent mean molar ellipticity of 6 acquisitions. Similar results were obtained with IAA- and NEM-alkylated b-rCLU (data not shown).

Extraction of inclusion bodies in solubilisation buffer containing 8 M urea yielded 100–120 mg protein/L bacterial culture, in which b-rCLU was by far the dominant protein present (data not shown). To remove minor impurities, solubilised inclusion bodies were subjected to SEC in a buffer containing 8 M urea and 5 mM DTT. Subsequent SDS-PAGE analysis of the purified b-rCLU showed a major species migrating at ∼45 kDa (Figure 1A), corresponding to the expected size of uncleaved, unglycosylated CLU, similar to the apparent mass of deglycosylated wtCLU in SDS-PAGE [14], [19]. However, once urea and DTT were removed by step-wise dialysis, HMW aggregates began to form (Figure 1B), presumably due to the progressive generation of inappropriate inter-molecular disulphide bonds between b-rCLU molecules. A minor band at ∼35 kDa was also detected by SDS-PAGE and Western blot analysis using anti-CLU antibodies (data not shown), which may represent a cleavage product of b-rCLU. Prior to dialysis of the solubilised inclusion bodies, when analysed by SEC in the presence of urea and DTT, b-rCLU migrated as a broad asymmetric peak with a maximum absorption at an elution volume of approximately 14 ml (∼450 kDa) (Figure 2A). After step-wise dialysis into PBS, although b-rCLU remained soluble, almost all the protein had formed HMW aggregates that ran at the size exclusion limit of the column (4×10^7^ Da); a minor peak at ∼18 ml (<67 kDa) probably represents minor proteolytic fragment(s) (Figure 2A). In contrast, as expected, wtCLU migrated as a mixture of oligomeric species [1], [14], [27], with multiple peaks eluting between 7.9 ml and 17 ml (between 4×10^7^ Da and 67 kDa) (Figure 2A).

It was found to be possible to reverse the aggregation of b-rCLU induced by removal of urea and DTT by strong reduction followed by cysteine capping or alkylation of free –SH groups. Although this was successful in substantively decreasing the size of species in solution (data not shown), circular dichroism (CD) spectroscopy indicated that all of these treatments induced considerable changes in secondary structure content (Figure 2B). The far UV CD spectra of b-rCLU that had been reduced and then alkylated or cysteine capped was significantly different to that of the starting material, which although inappropriately aggregated had a near-identical CD spectrum to wtCLU (Figure 2B).

**Figure 3:**
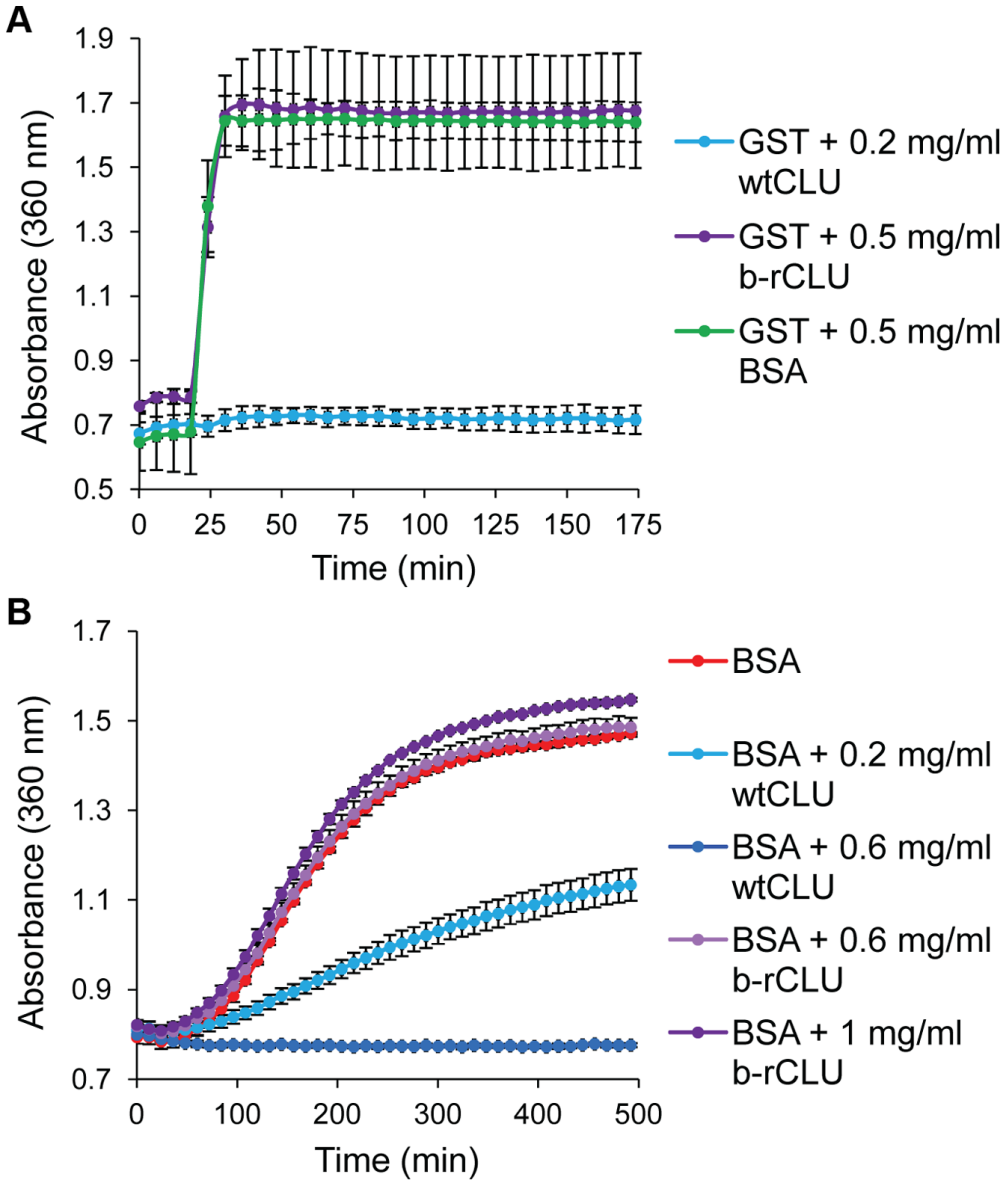
Chaperone assays for b-rCLU and wtCLU using 2 client proteins. (A) GST was heated at 60°C; BSA (negative control protein), wtCLU (positive control) and b-rCLU were tested at the concentrations indicated. The A_360_ is plotted at 6 min intervals (mean ± standard deviation; n  = 3). (B) BSA (1.5 mg/ml) was reduced with 20 mM DTT; wtCLU (positive control) and b-rCLU were tested at the concentrations indicated. The A_360_ is plotted at 12 min intervals (mean ± ½ range; n  = 2).

The chaperone activity of HMW b-rCLU was tested against two client proteins induced to aggregate using either heat (GST) or reductive stress (BSA) (Figure 3). Under all conditions tested, all forms of b-rCLU (including reduced and alkylated/capped forms; data not shown) lacked any detectable chaperone activity. In contrast, wtCLU dose dependently inhibited protein precipitation. As the bacterial expression system appeared incapable of producing a chaperone-active form of rCLU, a mammalian expression system was next investigated.

### Characterisation of rCLU Secreted by Transfected HEK293 Cells

**Figure 4:**
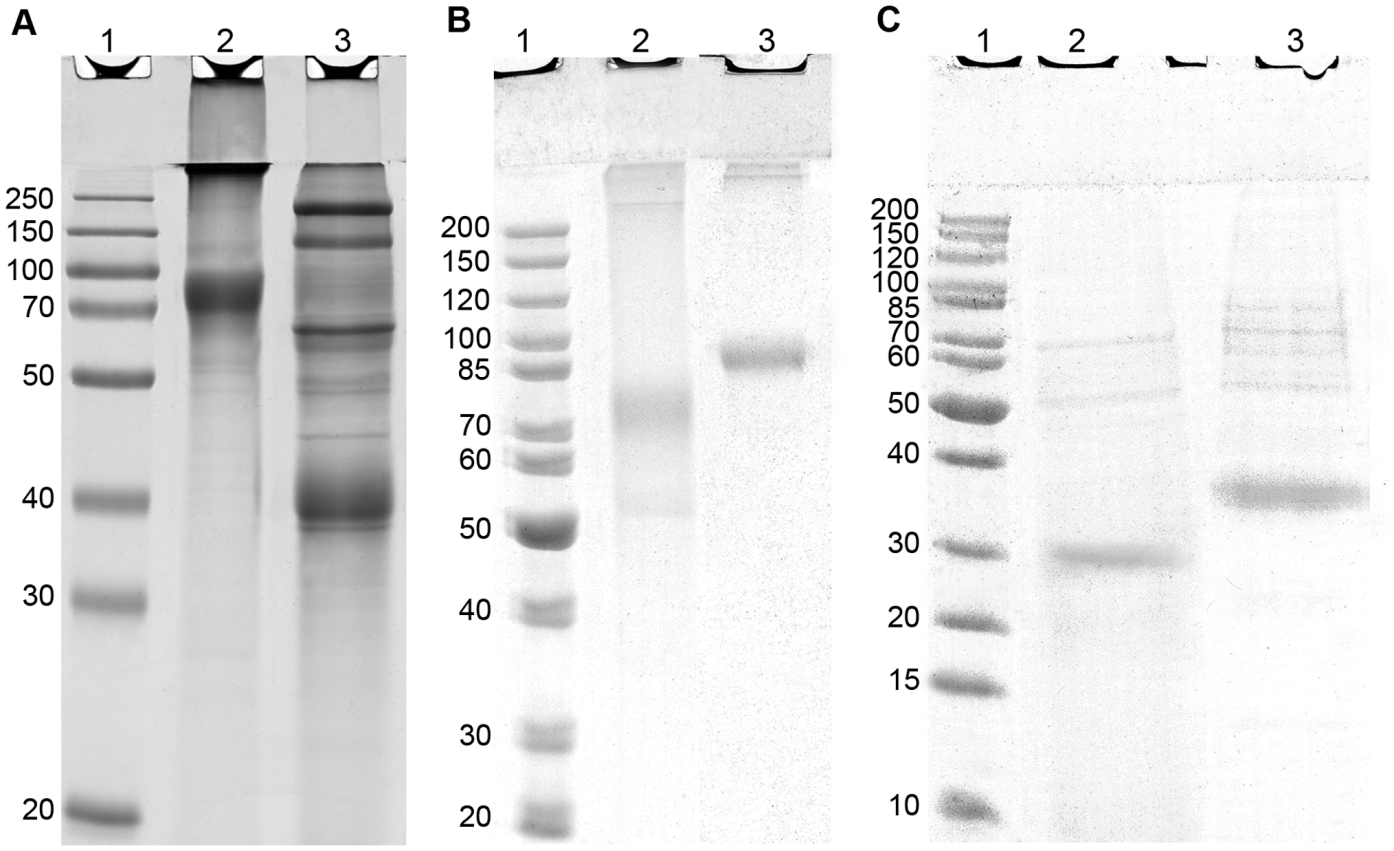
Images of Coomassie blue stained SDS-PAGE gels analysing m-rCLU secreted into FCS-supplemented versus serum-free media. (A) m-rCLU purified from culture medium containing 10% FCS, Lane 1: Molecular weight markers with size indicated in kDa; Lane 2: non-reducing; Lane 3: reducing conditions. (B) Non-reducing and (C) reducing SDS-PAGE analyses of m-rCLU purified from serum-free media and wtCLU. In (B) and (C), Lane 1: Molecular weight markers with size indicated in kDa; Lane 2: Purified m-rCLU; Lane 3: Purified wtCLU.

Compared to when transfected HEK293 cells were grown in medium containing FCS, the removal of FCS from the culture medium during expression significantly reduced the levels of HMW species in the purified sample (detected by non-reducing SDS-PAGE; Figure 4A, lane 2). Under reducing conditions, this HMW material dissociated into multiple bands of lower molecular weight, consistent with the reduction-induced dissociation of clusterin-client protein complexes (Figure 4A, lane 3). Similar analyses detected low levels of HMW material in m-rCLU purified from protein-free culture medium, but this was at comparably low levels to those detected in wtCLU purified from human plasma (Figure 4B). These HMW bands may represent SDS-resistant CLU oligomers or complexes formed between CLU and other proteins (as they react with anti-CLU antibodies in immunoblots; data not shown). Non-reducing SDS-PAGE analysis of m-rCLU detected a major band at ∼75 kDa and a minor band at ∼53 kDa (which may represent incompletely glycosylated CLU); wtCLU was detected as a major band at ∼85 kDa (Figure 4B). Reducing SDS-PAGE detected a band at ∼30 kDa for m-rCLU and at ∼35 kDa for wtCLU (Figure 4C); in both cases these bands correspond to the co-migrating α- and β-chains of CLU, indicating that m-rCLU was correctly post-translationally cleaved. The differences in mass between m-rCLU and wtCLU are most likely due to lower levels of glycosylation of m-rCLU by HEK293 cells. In both reduced samples minor bands between 50 and 85 kDa were present (Figure 4C), which may represent incompletely reduced forms and/or minor uncleaved species, which have been seen elsewhere [28].

**Figure 5:**
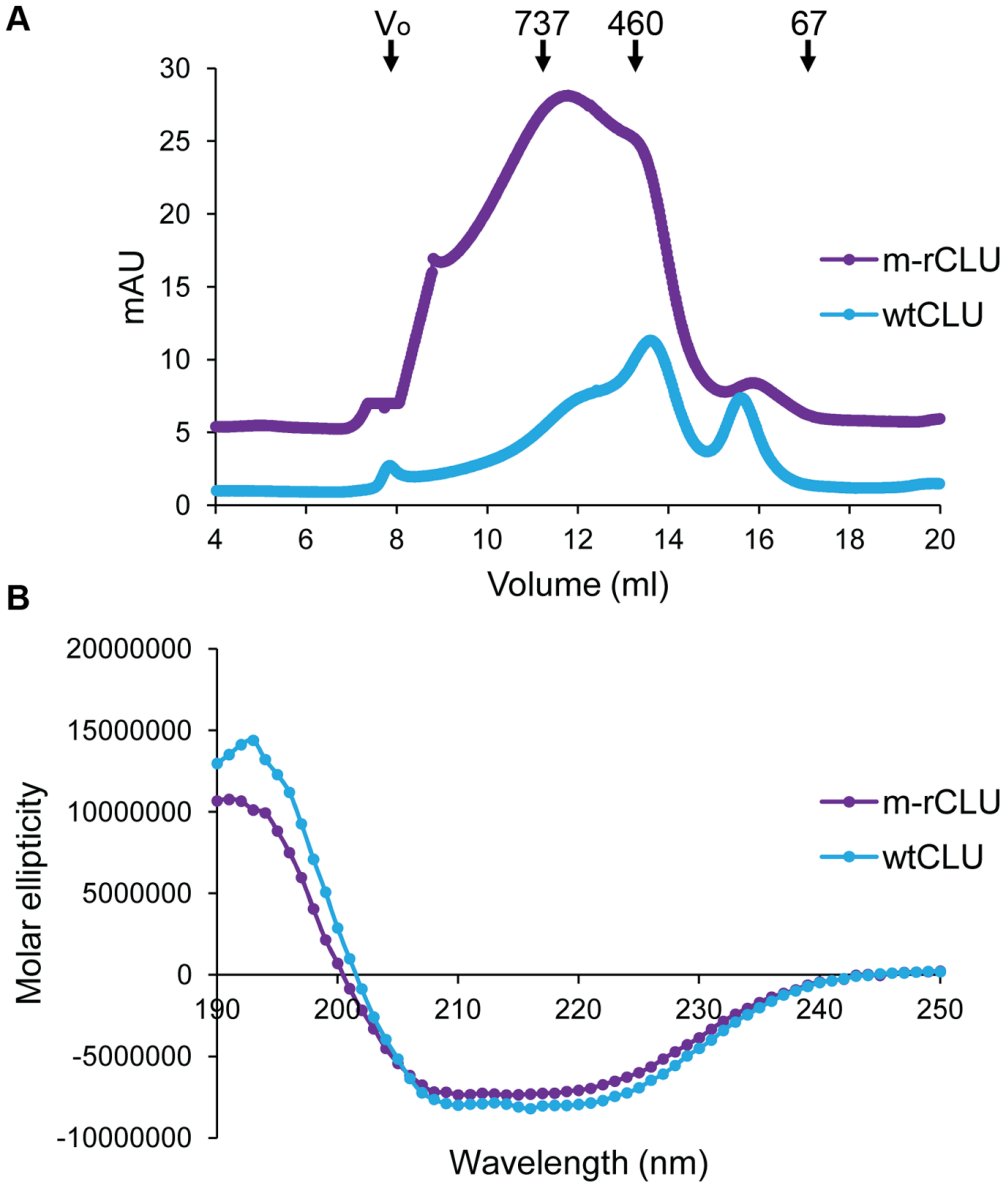
Superose™6 10/300 SEC separations and circular dichroism spectroscopy analysis of m-rCLU and wtCLU. (A) The Superose™6 10/300 column and samples were in PBS/Az prior to loading. The positions and masses (in kDa) of molecular weight markers are indicated by labelled arrows; the exclusion limit (V_o_) ≥4×10^7^ Da. The traces are deliberately vertically offset to more clearly display the results. (B) Plots represent mean molar ellipticity of 6 acquisitions.

SEC analysis of m-rCLU showed that it migrated as a broad peak between 7 and 15 ml (i.e. ranging from >1000 kDa down to <100 kDa), with a maximum absorbance at 11.7 ml (∼600 kDa) (Figure 5A). In contrast wtCLU showed a bias towards smaller species, with several peaks between 10 and 16 ml (i.e. ranging from ∼800 kDa down to ∼100 kDa) and a maximum absorbance at 13.6 ml (∼400 kDa). Other minor peaks were also detected for wtCLU at 15.5 ml and for m-rCLU at 15.9 ml (∼200 kDa) (Figure 5A). Thus m-rCLU and wtCLU span the same very broad size range of species in solution but m-rCLU contains more larger species than wtCLU. The far UV CD spectra of m-rCLU and wtCLU are very similar (Figure 5B), indicating that they are also similar in secondary structure content.

**Figure 6:**
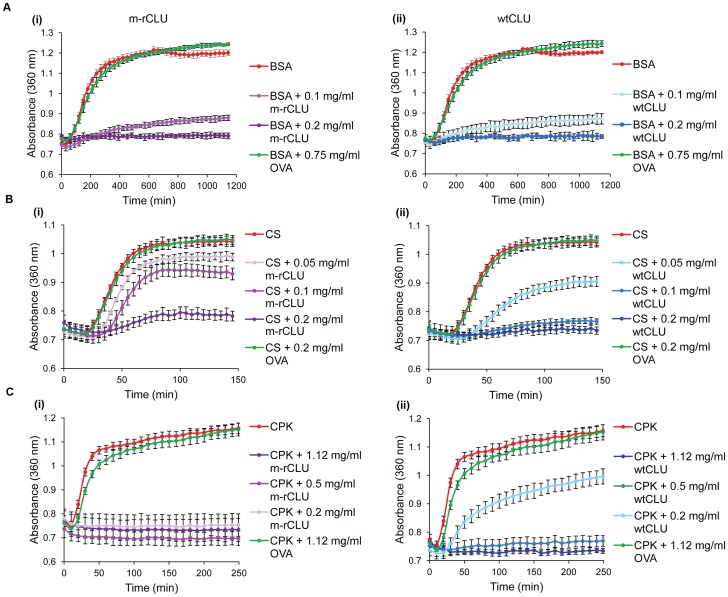
Chaperone assays of m-rCLU and wtCLU using 3 client proteins. (A) BSA (1.5 mg/ml) was induced to precipitate using 20 mM DTT. The A_360_ at 30 min intervals is plotted (mean ± standard deviation; n  = 3). (B) CS (200 µg/ml) and (C) CPK (1.12 mg/ml) were heated at 43°C to induce precipitation; the A_360_ (mean ± standard deviation; n  = 3) is plotted at intervals of 5 min (CS) or 10 min (CPK). In all cases, m-rCLU and wtCLU were tested at a range of concentrations and representative plots shown, and OVA was used as a negative control (at the highest concentration of CLU tested or greater).

The chaperone activity of m-rCLU was tested using a range of 3 client proteins. Like wtCLU, m-rCLU dose-dependently inhibited the reduction-induced precipitation of BSA; a concentration of 0.2 mg/ml (corresponding to a mass ratio of chaperone to client of 1∶7.5) fully inhibited precipitation of BSA (Figure 6A). Even at 0.75 mg/ml, the non-chaperone control protein OVA had no significant effect on the precipitation of BSA. Under these conditions, m-rCLU showed comparable chaperone activity to wtCLU at all concentrations tested (Figure 6A). In a second series of chaperone assays, like wtCLU, m-rCLU dose dependently inhibited heat-induced CS precipitation, though complete inhibition was not seen even at a 1∶1 mass ratio of m-rCLU to CS. Although m-rCLU was able to inhibit aggregation of CS, it was less effective than wtCLU against this client protein (Figure 6B). At 0.2 mg/ml wtCLU inhibited end-point precipitation of CS by 93% compared to 72% by m-rCLU; corresponding levels of inhibition for wtCLU and m-rCLU at 0.1 mg/ml and 0.05 mg/ml were 83% versus 29% and 46% versus 15%, respectively. A third series of chaperone assays were performed using CPK as the client protein (Figure 6C). Like wtCLU, m-rCLU dose dependently inhibited CPK precipitation. At a mass ratio of m-rCLU to CPK of 1∶5.6, m-rCLU fully inhibited the precipitation of CPK and was able to do this at a chaperone:client mass ratio approximately half that required for wtCLU (1∶5.6 versus 1∶2.24, respectively).

## Discussion and Conclusions

Bacterial expression systems typically produce high levels of recombinant products and are frequently used for the expression of proteins, however, they do not perform post-translational modifications. As the chaperone activity of CLU is not substantively affected by deglycosylation, we therefore investigated the potential to express rCLU in bacteria. CLU expressed in *E. coli* was found contained within the insoluble inclusion body fraction. To solubilise the b-rCLU contained in the inclusion bodies, 8 M urea and 10 mM DTT were required. Once solubilised, quick removal of the urea and DTT resulted in gross precipitation of b-rCLU from solution. Slower removal of these agents by step-wise dialysis maintained b-rCLU in solution but primarily as inappropriately disulphide-bonded HMW aggregates. It was possible to dissociate the HMW aggregates so formed by strong reduction, and to prevent their subsequent re-aggregation by alkylation (or cysteine capping) of reduced thiol groups on b-rCLU, but this had the consequence of inducing substantive differences in secondary structure content relative to wtCLU. Critically, all forms of b-rCLU produced that were soluble in physiological buffer (i.e. the absence of urea), including HMW b-rCLU and forms that had been strongly reduced and then alkylated or cysteine capped, lacked chaperone activity. Therefore another approach to produce functional rCLU was needed.

Previous work has shown that obtaining pure rCLU from mammalian cell expression systems is difficult [19]. Mammalian culture media proteins are subjected to sustained elevated temperature (37°C) and shear stress if the cultures are agitated. These conditions result in the progressive misfolding of some of the media proteins. The well-characterised chaperone action of CLU means that it will bind to these misfolded proteins to form soluble HMW complexes [1], [4], [8]. In the mammalian body these complexes are rapidly cleared [2], but in most cell culture systems these clearance mechanisms are not available, meaning that the HMW complexes remain in the medium and co-purify with rCLU. Previously, separating the HMW complexes from non-complexed rCLU proved difficult, and the yield was low [19].

We report here an optimised mammalian expression system using stably transfected HEK293 cells. The optimised method involved growing stably transfected HEK293 cells expressing m-rCLU in culture medium containing 10% (v/v) FCS, in which they grew satisfactorily and remained viable. To express m-rCLU, the cells were first passaged in DMEM:F-12 medium containing 5% (v/v) FCS, allowed to adhere to the culture surface for 16–24 h, and then cultured in FCS-free DMEM:F-12 medium for a subsequent 7–10 days before harvesting the culture supernatant. Removal of all protein from the end-stage culture medium was found to be essential to minimise the formation of stable high molecular weight chaperone-client complexes between clusterin and media proteins. Under these conditions 7.5–10 µg/ml m-rCLU was recovered from the culture supernatant (estimated by a semi quantitative immuno dot blot; data not shown). After 3-step purification (using G7 anti-CLU affinity chromatography, cation exchange chromatography and SEC) and concentration, 1 litre of tissue culture supernatant yielded ∼4.5 mg of pure, chaperone active m-rCLU. It may be possible to enhance this yield by simply increasing the density of HEK293 cells incubated in the protein-free medium (e.g. by using a suspension culture), but this remains to be confirmed.

Non-reducing SDS-PAGE analysis showed that m-rCLU migrated as a lower mass species than wtCLU (∼75 kDa versus ∼85 kDa, respectively). The apparent molecular weight of the α- and β-chains under reducing conditions was also smaller for m-rCLU (∼30 kDa) than wtCLU (∼35 kDa). This demonstrates that, like wtCLU, HEK293 expressed m-rCLU is a disulphide-bonded heterodimer comprised of α- and β-chains, and suggests that HEK293 cells glycosylate m-rCLU to a lesser extent than wtCLU. In fact, wtCLU purified from human plasma has been secreted by a variety of tissues, and thus represents a mixture of species with different types and levels of glycosylation [15] (but is probably dominated by clusterin secreted by the liver). HEK293 is an embryonic kidney cell line, and so will endow clusterin with its own unique level of glycosylation. Different levels of clusterin glycosylation is expected from differing cell types, as we have previously seen for clusterin expressed in yeast and insect cells ([17]; F Dawes, unpublished data).

CLU typically exists in physiological solution as a mixture of oligomers [1]; this characteristic of polydispersity is shared by a number of chaperones including the sHSPs [29]. As has been previously shown by Stewart *et al*. (2007) and in this study, when analysed by SEC a small fraction of wtCLU ran at the size exclusion limit of the column (≥4×10^7^ Da), and a major peak was detected at ∼400 kDa. In this study, compared to wtCLU, the forms of m-rCLU in solution were biased towards higher molecular weight species. Deglycosylated CLU has previously been shown to migrate in SEC as a broad peak with a maximum at ∼900 kDa [14]. Thus, the lower levels of glycosylation of m-rCLU, relative to wtCLU, may have the effect of increasing the mean size of oligomers in solution, but this remains to be definitively established. In the human body wtCLU is produced by many different types of tissues/cells and typically blood from more than one donor is pooled and used to purify wtCLU, leading to a sample with heterogeneous glycosylation levels.

CD analysis showed that m-rCLU had similar secondary structure content to wtCLU, as expected. It was previously shown that deglycosylated CLU has a similar secondary structure content to wtCLU [14]. HEK293 expressed m-rCLU was chaperone active against all three client proteins tested. Depending on the individual client protein, m-rCLU showed either the same, increased or decreased ability to inhibit precipitation compared to wtCLU. These client protein-dependent differences in relative chaperone activity may relate to differences in glycosylation and/or solution size between m-rCLU and wtCLU, although further work would be required to confirm this.

HEK293 cell expressed m-rCLU is cleaved into α- and β-chains, glycosylated, has a similar secondary structure content to wtCLU, and is chaperone-active. Therefore, this report provides the first description of a viable method to produce structurally and functionally validated rCLU that can in future be used to: (i) express and characterise CLU mutants, to identify residues important in structure and function, (ii) metabolically label CLU for structural studies (e.g. NMR), and (iii) produce large quantities of rCLU for use as a therapeutic agent to treat a wide range of protein deposition diseases, or other conditions. It is thought that a primary physiological role for CLU is as an extracellular chaperone which stabilises misfolded extracellular proteins and facilitates their clearance from the body. Thus the development of a robust source of correctly processed, functionally active rCLU opens the door to increased understanding of how CLU performs its physiological roles and potentially gives us the ability to develop this new knowledge towards an outcome that will enhance human health.

## Supporting Information

Figure S1 **Diagram depicting methods trialled to produce rCLU in (i)** ***E. coli*** **and (ii) HEK293 cells.** (i) Three methods were tested in an attempt to produce soluble b-rCLU (in PBS) after extraction from inclusion bodies; a) direct dilution, b) direct dialysis, and c) step-wise dialysis. Red crosses indicate where b-rCLU precipitated from solution. Further tests (indicated) were undertaken with soluble b-rCLU obtained following step-wise dialysis, the results indicating that the product was misfolded and lacked chaperone activity. (ii) A mammalian expression system, using HEK293 cells, was tested using both culture medium containing foetal calf serum (FCS) and protein free culture medium. Expression of m-rCLU using protein free culture medium enabled the production of pure, chaperone-active protein that was correctly folded and post-translationally processed.
(TIF)
