# Supplementary figures and images for: Expression and Purification of Chaperone-Active Recombinant Clusterin
Source: PLoS One. 2014 Jan 23;9(1):e86989. doi: 10.1371/journal.pone.0086989 (PMC3900688; doi:10.1371/journal.pone.0086989)

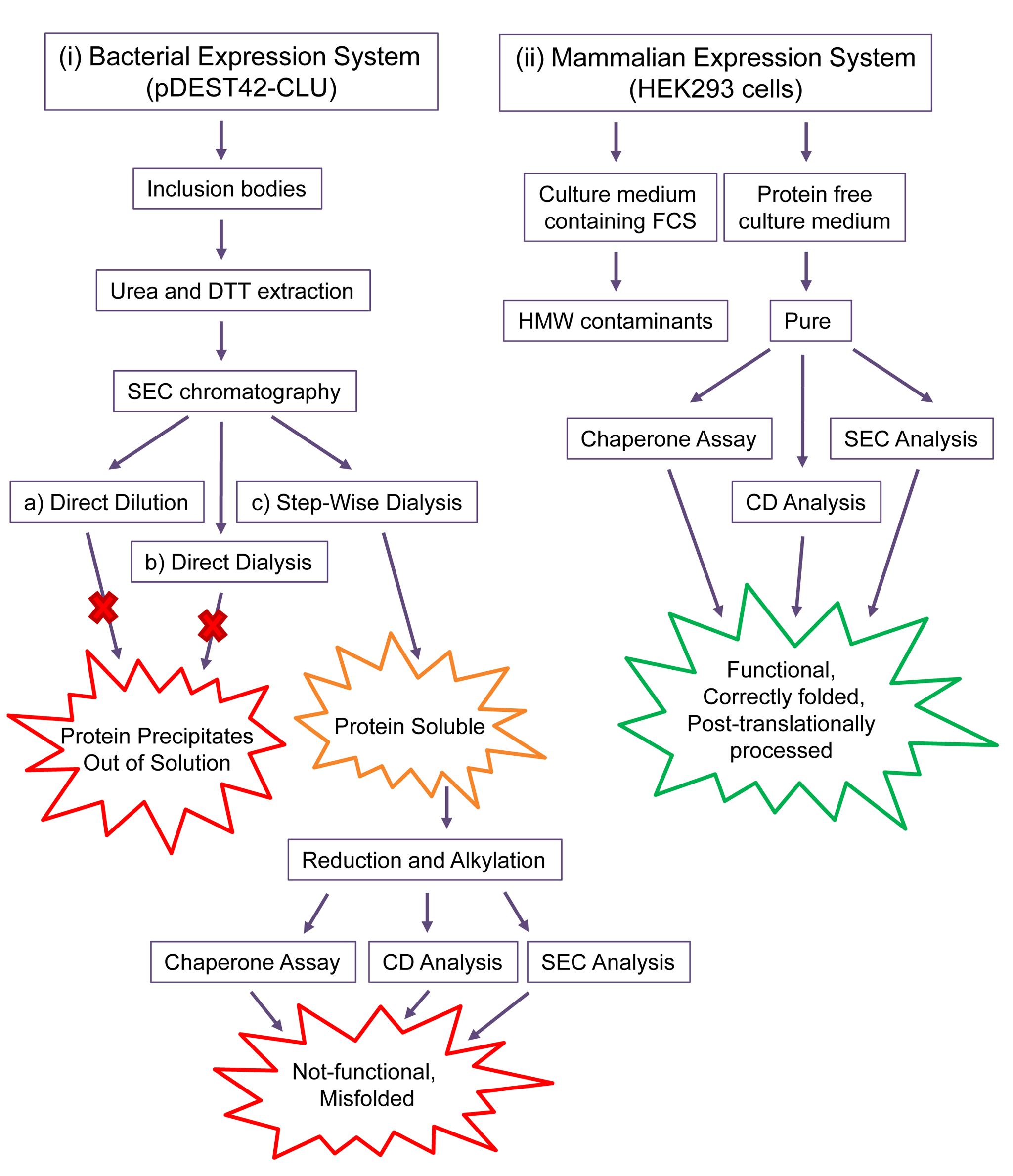

Supplement: Figure S1 — Diagram depicting methods trialled to produce rCLU in (i) E. coli and (ii) HEK293 cells. (i) Three methods were tested in an attempt to produce soluble b-rCLU (in PBS) after extraction from inclusion bodies; a) direct dilution, b) direct dialysis, and c) step-wise dialysis. Red crosses indicate where b-rCLU precipitated from solution. Further tests (indicated) were undertaken with soluble b-rCLU obtained following step-wise dialysis, the results indicating that the product was misfolded and lacked chaperone activity. (ii) A mammalian expression system, using HEK293 cells, was tested using both culture medium containing foetal calf serum (FCS) and protein free culture medium. Expression of m-rCLU using protein free culture medium enabled the production of pure, chaperone-active protein that was correctly folded and post-translationally processed. (TIF) [file pone.0086989.s001.tif]
